# Reduced incidence of end stage renal disease among the elderly in Denmark: an observational study

**DOI:** 10.1186/1471-2369-13-131

**Published:** 2012-10-03

**Authors:** James G Heaf, Sonja Wehberg

**Affiliations:** 1 Herlev Hospital, University of Copenhagen, Graevlingestien 9, 2880, Bagsvaerd, Denmark; 2 Research Unit of Clinical Epidemiology, Centre for National Clinical Databases – South, University of Southern Denmark and Odense University Hospital, Odense, Denmark

**Keywords:** ESRD, Epidemiology, Uremia progression, Hypertension, Antihypertensive therapy, ACE inhibition

## Abstract

**Background:**

A number of studies during the nineties have shown that antihypertensive therapy, particularly using RAS blockade, can reduce uremia progression, and ESRD incidence.

**Methods:**

National incidence rates were studied of end stage renal disease (ESRD) for Denmark between 1990 and 2011, and of national prescription of antihypertensive drugs between 1995 and 2010, in order to investigate whether prescription rates had changed, and whether the expected change in ESRD had materialized. The Danish Nephrology Registry (DNR) is incident and comprehensive. Incidence rates were classified according to renal diagnosis.

**Results:**

ESRD incidence was constant for age groups <60 years. Incidence rates rose during the nineties for all cohorts >60 years. Since 2001 rates for subjects 60–70 years have fallen from 400 ppm/yr to 234, and since 2002 for subjects 70–80 years from 592 to 398. The incidence of patients >80 years has increased to 341. The falling incidence for patients 60–80 years was distributed among a number of diagnoses. Since 1995 national antihypertensive drug therapy has increased from 24.5 defined daily doses (DDD)/citizen/yr to 101.3, and the proportion using renin-angiotensin system (RAS) blockade from 34 to 58%.

**Conclusions:**

This national study has shown a reduction in actively treated ESRD incidence among patients aged 60–80 years. It is possible that this is the result of increased antihypertensive prescription rates, particularly with RAS blockade. If it is assumed that therapeutic intervention is the cause of the observed reduced incidence, ESRD incidence has been reduced by 33.8 ppm/yr, prevalence by 121 ppm, and ESRD expenditure by 6 €/citizen/yr.

## Background

During the nineties, a number of studies were published, showing that antihypertensive therapy in patients with chronic kidney disease (CKD) delayed the progression of uremia. Whether intensive antihypertensive therapy, with the aim of reducing blood pressure to below 130/80 (as opposed to conventional therapy) is *per se* effective, is still controversial. The original MDRD study [1], comparing low and high intensity antihypertensive therapy, found that beneficial effects were limited to patients with proteinuria >1 g/day, and other large studies, such as the REIN-2 [2] and AASK [3] trials, have failed to demonstrate an overall effect of intensive treatment. However, the ESCAPE trial of 385 children with CKD, demonstrated a 35% reduction in uraemia progression. Anithypertensive therapy, regardless of type, reduces proteinuria [1,3]. In contrast, there is no doubt that renin-angiotensin system (RAS) blockade has a specific protective effect over and above other antihypertensive agents, presumably because of its extra anti-inflammatory and anti-proteinuric effects. Early studies suggested that uraemia progression could be delayed by 35% [4,5]. Since then a number of large studies have shown that RAS blockade prevents the development of diabetic nephropathy [6] and reduces uraemia progression by 30-40% [7-10]. These studies have recently been reviewed [11]. The effect is present both in proteinuric and non-proteinuric diseases, but the effect is greater, the greater the degree of proteinuria and the achieved reduction in proteinuria. We hypothesized that these papers will have led to national antihypertensive prescription changes which would in turn result in a reduction in the incidence of end stage renal disease (ESRD).

## Results

While the national population only rose by 8.3% during the study period, considerable changes in the age structure were seen. The population aged 60–69 years increased from 492,000 to 683,000 (38.9%), 70–79 years from 367,000 to 386,000 (5.3%), and over 80 from 188,000 to 227,000 (21.5%).

**Figure 1:**
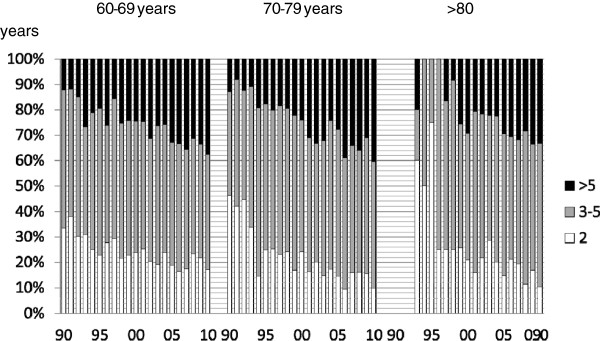
**Changes in Charlson Comorbidity Index over time,according to age group**.

The age of the oldest incident ESRD patient in the registry rose almost linearly from 75.3 years in 1985 to 81.8 in 1990 and 95.1 in 2007, after which it stabilized, an average increase of 7.6 months/year. The average age rose from 51.6 ±15.9 to 61.9 ± 16.1 in 2001 and to 64.5 ±18.2 years in 2010. The Charlson Comorbidity Index remained steady for patients <50 years, but increased significantly for older age groups (Table 1, Figure 1).

**Table 1:** Average Charlson Comorbidity Index and age: time trends

	**Year**				
**Age**	**1990**	**2000**	**2010**	**Correlation coefficient**	**Significance**
0-19	2,00 ±0,2^a^	2,17 ±0,4	2,13 ±0,4		NS
20-29	2,97 ±1,3	3,32 ±1,5	2,59 ±1,2		NS
30-39	3,96 ±1,6	3,36 ±1,6	3,33 ±1,9		NS
40-49	3,18 ±1,7	3,53 ±1,6	3,44 ±1,8		NS
50-59	3,47 ±2,0	4,32 ±2,1	4,22 ±2,2	0,11	<0.001
60-69	3,51 ±1,5	4,24 ±2,0	5,01 ±2,4	0,15	<0,001
70-79	3,36 ±1,8	4,20 ±1,9	5,08 ±2,0	0,21	<0,001
≥80	2,67 ±0,8^b^	4,56 ±2,5	4,86 ±2,0	0,17	<0,001

**Figure 2:**
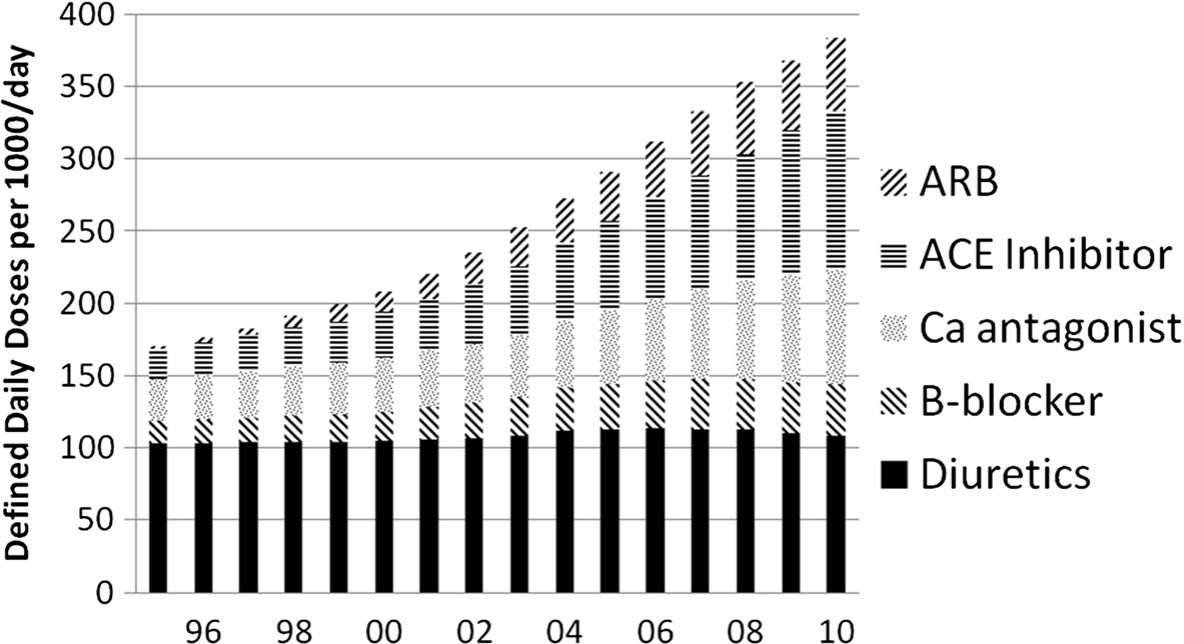
**National consumption of the commonest antihypertensive agents 1995–2010**.

Data concerning national prescription rates for the four most common antihypertensive drugs were available for 1995–2010 (Figure 2). During this period consumption rose from 24.5 to 101.3 DDD/capita/year, a four-fold increase. The proportion of consumption attributed to renin-angiotensin system (RAS) blockers rose from 34 to 59%.

**Figure 3:**
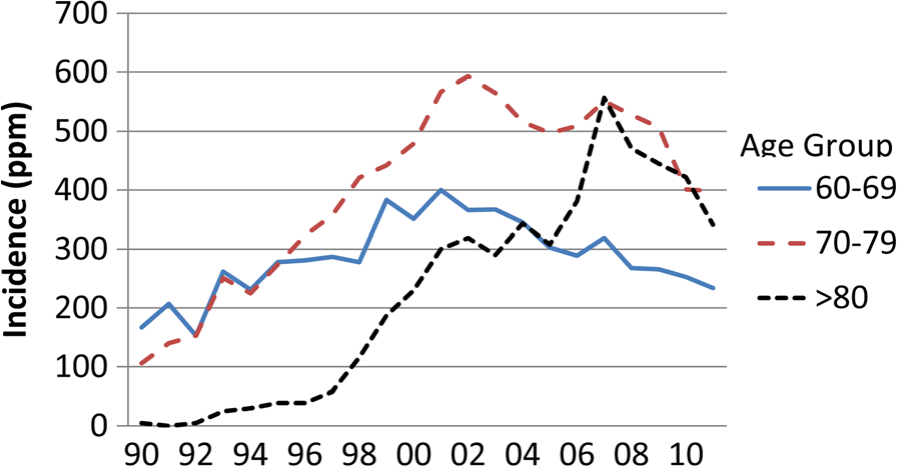
**Incidence of ESRD in patients>60 years 1990–2011**.

The incidence of ESRD is shown in Table 2, and the absolute patient numbers in Table 3. The incidence of patients <60 years remained approximately constant during the entire period of observation (Table 2). A fall in incidence of 19% between 2001 and 2011 for this group was not significant. Since 2001 the ESRD incidence has fallen by 7% among 40–49 year olds, and 26% among 50-59-year-olds. These changes were not significant. The incidence of patients 60–70 years (Figure 3) rose from 167 patients per million of population (ppm) in 1990 to 400 ppm in 2001, and then fell steadily to 234 ppm, a 42% fall (p < 0.01). The incidence of patients 70–80 years rose from 106 ppm in 1990 to 593 in 2002, and then fell to 398, a 33% fall (p < 0.03). Active treatment of patients over 80 years was virtually nonexistent at the beginning of the study period, rose slightly to 58 ppm in 1997, and then rose rapidly to 557 ppm in 2007. It has since fallen to 341 ppm, a 39% fall (NS). The secular trend for incidence for the years 2000–2011 was significant for those aged 60–70 (r = −0.91, p < 0.001), but not for patients 70–80 years (r = −0.45, NS).

**Table 2:** Incidence of ESRD 1990–2011 according to age, and standardized rate (patients per million, ppm)

	**Age (years)**								**Standardised**	**Population (thousand)**
**Year**	**0-19**	**20-29**	**30-39**	**40-49**	**50-59**	**60-69**	**70-79**	**≥80**	Rate (ppm)*	
1990	6	45	32	101	112	167	106	5	64	5,135
1991	9	34	43	75	156	207	140	0	71	5,146
1992	11	24	64	69	150	153	153	5	67	5,162
1993	14	34	56	106	182	262	251	25	95	5,181
1994	7	38	80	71	145	231	225	30	84	5,197
1995	15	47	53	104	136	278	274	39	96	5,216
1996	9	35	60	89	145	281	323	39	96	5,251
1997	8	34	70	86	167	287	357	58	103	5,275
1998	14	32	50	98	162	278	421	116	108	5,295
1999	6	31	64	105	150	383	442	187	122	5,314
2000	10	26	67	120	186	351	479	230	129	5,330
2001	11	28	49	90	191	400	567	300	137	5,349
2002	9	22	60	80	143	366	593	319	130	5,368
2003	6	43	43	96	152	367	565	290	131	5,384
2004	14	35	45	110	153	345	516	343	130	5,398
2005	11	16	48	73	176	303	497	308	117	5,411
2006	13	21	41	84	133	289	509	381	117	5,427
2007	13	26	63	94	184	319	551	557	140	5,447
2008	10	29	32	85	146	268	528	471	120	5,476
2009	16	49	45	82	167	266	507	445	125	5,511
2010	6	27	53	70	154	253	401	422	108	5,535
2011	5	22	53	84	141	234	398	341	102	5,561

*Based on population structure in 1990.

**Table 3:** Incidence of ESRD 1990–2011 according to age (absolute numbers)

	**Age (years)**								**Total**
**Year**	**0-19**	**20-29**	**30-39**	**40-49**	**50-59**	**60-69**	**70-79**	**≥80**	
1990	9	36	24	77	60	82	39	1	328
1991	11	27	32	58	85	100	52	0	365
1992	14	19	48	55	83	73	57	1	350
1993	17	28	42	83	104	124	93	5	496
1994	9	30	61	56	86	108	83	6	439
1995	18	37	41	81	83	129	101	8	498
1996	11	28	47	68	96	130	119	8	507
1997	11	26	56	66	113	134	131	12	549
1998	17	25	42	72	114	130	154	24	578
1999	8	24	53	79	108	182	161	39	654
2000	12	21	55	91	138	168	174	48	707
2001	14	20	41	67	144	195	204	63	748
2002	11	15	49	61	108	183	210	69	706
2003	8	31	37	72	116	189	200	63	716
2004	19	23	36	85	116	185	182	75	721
2005	14	11	40	57	133	170	174	68	667
2006	17	13	32	68	99	170	179	85	663
2007	18	16	48	75	134	198	197	125	811
2008	14	18	24	69	105	172	193	106	701
2009	21	33	34	67	121	179	188	102	745
2010	8	19	41	60	112	175	157	104	676
2011	7	14	39	68	101	160	154	78	621

The contributions of risk reduction population structure changes and population number changes is shown in Table 4. For all groups, a fall in incidence was noted, which was independent of changes in population number and structure. The fall was greatest for patients 60–80 years.

**Table 4:** RiskDiff analysis of contributing factors to changes in incidence 2001 vs. 2011

	**ESRD incidence (p.p.m)**		**Total change**		**Contributing factor**					
					**Change in risk**		**Change in population structure**		**Change in population size**	
**Age group (years)**	**2001**	**2011**	**No.**	**%**	**No.**	**%**	**No.**	**%**	**No.**	**%**
0-110	140	112	−127	−17.0	−213	−28.5	62	8.3	24	3.2
0-80	133	102	−142	−20.7	−223	−32.6	61	8.9	20	2.9
0-60	67	54	−57	−19.9	−53	−18.4	−3	−1.0	−1.5	−0.5
60-80	472	294	−85	−21.3	−142	−35.6	−9	−2.2	66	16.5

In order to analyze which renal diagnoses had experienced the highest fall in incidence, incidence rates for the years 2000–01 were compared with the years 2009–2010 for common renal diagnoses. The results are shown in Table 5. Improvements were seen over a wide range of diagnoses, with no clear distinction between proteinuric (e.g. diabetic nephropathy and glomerulonephritis) and non-proteinuric (e.g. CIN and polycystic renal disease). A post-hoc analysis of sub-diagnoses in the diagnosis group “Other”, showed falls in most subgroups, but the number of patients in each subgroup did not permit statistical analysis. However, the incidence of patients with vasculitis (ICD-10 codes M30.x and M31.x) among patients aged 60–79 fell 58% from 20.8 ppm/year to 8.7 (p < 0.03).

**Table 5:** Incidence of ESRD (ppm/year) in 2000–01 compared to 2009–10, according to age group and renal diagnosis

**Renal diagnosis**	**Age group**	**2000-01**		**2009-10**		**% Change**	**Sig.**
		**No.**	**Incidence**	**No.**	**Incidence**		
Small	60-69	72	74.5	94	70.7	−5	NS
	70-79	119	165.3	100	134.0	−19	NS
	Combined	191	113.3	194	93.4	−18	NS
Glomerulonephritis	60-69	41	42.4	35	26.3	−38	NS
	70-79	16	22.2	17	22.8	3	NS
	Combined	57	33.8	52	25.0	−25	NS
Chronic Interstitial	60-69	48	49.7	44	33.1	−34	NS
	70-79	49	68.1	34	45.6	−33	NS
	Combined	97	57.5	78	37.6	−35	NS
Polycystic	60-69	25	25.9	21	15.8	−39	<0.05
	70-79	12	16,7	11	14.7	−12	NS
	Combined	37	21.9	32	15.4	−30	NS
Hypertensive	60-69	40	41,4	32	24.1	−42	NS
	70-79	46	63,9	53	71.0	11	NS
	Combined	86	51.0	85	40.9	−20	NS
Type 1 DM	60-69	28	29.0	20	15.0	−48	NS
	70-79	15	20.8	11	14.7	−29	NS
	Combined	43	25.5	31	14.9	−42	NS
Type 2 DM	60-69	53	54,9	71	53.4	−3	NS
	70-79	51	70,8	62	83.1	17	NS
	Combined	103	61.7	133	64.1	4	NS
Other	60-69	59	61.1	31	23.3	−62	<0.002
	70-79	70	97.2	52	69.7	−28	NS
	Combined	129	76.5	83	40.0	−48	<0.001
Total	60-69	366	378.9	348	261.7	−31	<0.001
	70-79	378	525.0	340	455.8	−13	NS
	Combined	744	441.3	688	331.4	−25	<0.001

## Discussion

The incidence of actively treated ESRD has increased continuously since the start of maintenance dialysis therapy in the sixties. This has been largely driven by a steady increase in take-on rates. There are two reasons for this. Firstly, economic growth and a steady reduction in the costs of dialysis, mean that health services have been able to afford treating more patients. Secondly, the results of treating patients of increasingly high age and morbidity, in particular DM, have improved, such that active treatment of marginal groups is justified. This pattern can be seen in this national survey. During the period, a linear increase in the age of the oldest patient in the registry was seen, and the age-adjusted morbidity increased. This increase, approximating 6 months per year, was far greater than the increase in expected life expectancy of the background population. During the nineties, increased incidence rates for 60-80-year-olds and diabetics [12] were seen. After 2000, in response to encouraging treatment results [13], the incidence of 80-year-olds rapidly increased, while the incidence of diabetics stabilized [12]. Thus, it is highly likely that the initial rise in incidence in elderly age groups is an expression of these secular trends. The increase in incidence rates for Type 2 diabetics over the age of 70 after 2001 is probably also part of this trend. One would therefore expect that incidence rates at some point would stabilize at a higher level.

The present study shows that ESRD incidence among patients <40 years has remained stable for 20 years. Since 2001 the ESRD incidence has fallen by 7% among 40–49 year olds, and 26% among 50-59-year-olds. These changes were not significant. Since 2001 the ESRD incidence among 60-70-year-olds has fallen by 42%, and 70-80-year-olds since 2002 by 33%. The Riskdiff analysis (Table 4) shows that these changes are independent of population structure. A recent fall in incidence since 2007 of 39% among patients older than 80 years is too recent to be meaningful. It could be due to a real increase in incidence similar to the 60–80 year-olds, or a waning enthusiasm by primary health carers for referring these often frail patients for treatment. This is in contrast with other national results [14,15] which have merely showed a stabilization in these age groups. We believe this to be one of the first cases of a national reduction in ESRD incidence in the absence of social or economic unrest. As a direct result the number of prevalent dialysis patients in Denmark has now fallen by 5% since 2008. Taiwan has also noted a fall in ESRD incidence from 432 to 384 ppm between 2005 and 2008; this has not however yet resulted in a fall in prevalence [15].

During the nineties, a number of possibly modifiable factors in the progression of uraemia were identified. Aggressive treatment of hypertension is probably important [1,16]. RAS blockade by ACE-I and A2A were shown to have specific nephroprotective properties, in particular in patients with diabetes and proteinuria [4,5,9,10,17,18]. A protective effect of protein restriction has been suggested [19], as has a nephrotoxic effect of tobacco [20]. Increased use of these prophylactic measures would be expected to reduce ESRD incidence. Figure 2 shows that the prevalence of antihypertensive therapy, and in particular RAS blockade in the general population has indeed increased substantially, to a level of 0.27 DDDs/capita/year. The percentage of non-smokers (or irregular) has increased from 58 to 77% between 1995 and 2008 [7]. There is no evidence that the incidence of hypertension has increased in Denmark; thus the increased drug use is probably an expression of more intensive individual therapy. This study has only documented an increase in general antihypertensive therapy, in particular RAS blockade, and a reduction in ESRD incidence among the elderly. This being an observational study, any discussion about causality must be purely speculative, but it is possible that the observed decrease in ESRD incidence is an expected consequence of the intensified prophylaxis. If this is so, there appears to be a lag time of at least 5 years between a change in antihypertensive therapy and a decrease in incidence. This is not surprising: in order to delay ESRD significantly, treatment has to be initiated while the patient still has a significant renal function. During the period of observation, there were no governmental changes in the organization or financing of ESRD treatment. All patients are treated at hospital-based, publicly financed nephrology centers. There has been an increased awareness of the importance of predialysis nephrology care, and today all patients with a GFR below 30 ml/min are recommended specialist care. This may have contributed to the fall in ESRD rates, independently of concurrent antihypertensive therapy and RAS blockade. In common with international trends, there has probably been a tendency to start dialysis at a higher level of GFR since 2000; this would *a priori* increase the number of ESRD patients slightly.

Considerable changes have occurred in the background population between 1990 and 2011. The average longevity has increased from 72.2 to 77.8 years for males, and 77.3 to 81.6 for females [21]. While increased longevity will of course be expected to increase the absolute numbers of elderly patients, it will not in itself affect the incidence, expressed as a fraction of the population at risk. The Riskdiff analysis shows that the observed changes are real and independent of any change in population structure. It shows that the evolution of the population 60–80 years would have lead to a rise of incidence of 16% while the observed incidence was −21%. The underlying risk fell by 36%. Ischaemic heart disease as a cause of death fell from 25.6% of all deaths to 9.2%, and cerebrovascular disease from 9.1% to 6.9%. These changes could also partly be related to more intensive antihypertensive therapy. It is difficult to predict how these changes might affect ESRD incidence: on the one hand, since cardiac and renal disease are often related, with common etiologies such as diabetes, atherosclerosis and hypertension, a better cardiac survival might lead to more patients surviving to renal failure; on the other hand, the prophylactic treatments that reduce the incidence of heart disease might also reduce the incidence of renal disease.

Two findings were surprising. The fall in incidence was distributed between different renal diagnoses, without any clear distinction between proteinuric and non-proteinuric diseases. A change in coding practices during the period of observation cannot be excluded, but since the diagnoses were made by a small group of nephrologists, using standard ERA-EDTA definitions, we consider this unlikely. Also, the fall in incidence was mainly confined to patients over the age of 60. While ESRD incidence was lower for patients aged 30–59 was lower in 2011 than 2001, the difference was smaller and non-significant, partly because of the small number of patients in these age groups. There are several possible explanations for this apparent difference: some diagnoses, common among younger patients, such as polycystic renal disease and hereditary disorders, may be less amenable to prophylaxis; early diagnosis and prophylaxis may be rarer among younger patients; it is possible that long-term therapy is required to make a noticeable difference. Antihypertensive therapy has been shown in the ESCAPE study to also be effective in children [16]. This is a recent study, which cannot have affected previous therapy; no data is available concerning antihypertensive therapy among children in this population. A further disadvantage of this study is that data concerning antihypertensive use was only available after 1995, and only as DDDs, rather than number of patients being treated.

Not all health indicators have moved in the right direction. The number of obese adults (body mass index >30 kg/m^2^) has risen from 5.5% in 1987 to 7.6% in 1994 and to 13.4% in 2010. It is thus all the more remarkable that the expected epidemic in diabetic nephropathy has not occurred, and that the incidence of type 2 diabetic nephropathy is stabilised. Thus, the theory that intensive prophylactic intervention can reduce the incidence of diabetic nephropathy seems to have been justified in practice.

It is possible that unidentified factors could have contributed to the fall, e.g. a reduction in consumption of nephrotoxic drugs or an improvement in the urological treatment of patients with post-renal uremia. Improvements in immunosuppressive therapy may have contributed to the fall in vasculitis incidence. It is even possible that the initial increase in incidence seen among patients over 70 years is partly caused by a postponement of ESRD among patients 60–70 years to a later age, the real fall in incidence thereby being exaggerated.

If one assumes that there is a causal connection and that, without intervention, incidence among 60-70-year-olds would have remained at 400 ppm and among 70-80-year-olds at 592, a rough estimate of the possible economic benefits of prophylaxis can be made. Further assumptions are required for this calculation: the mean survival after ESRD is 4.3 and 2.5 years respectively (DNR average 2000–2010); the average cost is €50,000/year. It then follows that ESRD incidence has been reduced by 33.8 ppm/year, ESRD prevalence by 121 ppm and ESRD expenditure by approximately €6 per capita/year. Drug expenditure needs to be subtracted from this to calculate the net economic benefit. For commonly used ACE inhibitors this is however less than 10 cents/day.

## Conclusions

During the period of observation, a significant fall in ESRD incidence in the population between 60–80 years age was seen, and also an increase in prescription rates for antihypertensive drugs, particularly RAS blockade. It is possible that these two phenomena are connected. The findings suggest that ESRD is a preventable disease.

## Methods

All patients resident in Denmark, and thus possessing a national identity number, starting active treatment for ESRD between 1.1.1990 and 31.12.2011 were included in the study. Their data were extracted from the following databases:

1) The Danish Nephrology Registry (DNR) contains data from all patients starting active treatment in Denmark. The database is incident, prospective and has been comprehensive since 1.1.1990. Cross-checks with performed dialyses registered in the National Patient Registry show that >99% of all patients with ESRD are included. A patient is regarded as having ESRD if (a) the nephrologist considers him/her to have ESRD on the day of first active treatment or later; (b) a renal transplant is performed; (c) there is some doubt regarding the reversibility of the uraemia (e.g. crescentic glomerulonephritis, acute tubulointerstitial nephropathy), but the patient has received at least 90 days of dialysis. A recent quality assessment study has shown that the risk of not being included in the registry is less than 1% ( [22]). Patient sex, renal diagnosis, and age at ESRD were noted.

2) Discharge diagnoses for all admissions to hospital between 1977–2010 were extracted from the National Patient Registry (LPR). The Charlson Comorbidity Index (CCI) [23] at ESRD was calculated. All patients received two CCI points for uremia/ESRD regardless of whether they had previously been admitted for this condition.

3) National population statistics were extracted from the National Population Registry (Statistics Denmark).

4) National prescription rates for antihypertensive drugs, excluding diuretics, between the years 1995–2010 were extracted from the Danish Medicines Agency. Prescriptions were classified as β-blockers, calcium antagonists, angiotensin converting enzyme inhibitors (ACE-I) and angiotensin receptor blockers (A2A). Consumption of other antihypertensive classes was minimal. Consumption was assessed as number of defined daily doses (DDD) 1000 citizens/day.

ESRD incidence rates were calculated for different age groups and renal diagnoses. Renal diagnoses up to 31.12.2010 were categorized as shrunken kidneys (ICD-10 code N18.x, Q60.5), chronic glomerulonephritis (N02.x-N07.x), chronic interstitial nephritis (CIN) (N11.x-N15.x, N20.x, N31.9, Q62.x), polycystic renal disease (Q61.x) hypertensive (I12.x), type 1 diabetic (DM) (E10.x), type 2 diabetic (E11.x), other.

### Statistics

Incidence rates between years were compared using the χ^2^ test. Secular trends were analysed using Pearsson product–moment correlation.

A post-hoc analysis was performed to compare incidence rates between 2001 and 2011. The RiskDiff program [24] is a tool can be useful to study the differences in the incidence or mortality observed in two given situations (such as time points, geographical areas, or males versus females). The method performed splits the observed differences into three components: (1) the risk itself, (2) difference attributed to changes in the population size and (3) difference attributed to changes in population structure.

## Abbreviations

ACE: Angiotensin converting enzyme; A2B: Angiotensin 2 receptor blockers; CCI: Charlon Comorbidity Index; CKD: Chronic Kidney Disease; DDD: Defined daily dose; DM: Diabetes mellitus; DNR: Danish Nephrology Registry; ESRD: End stage renal disease.

## Competing interests

The author(s) declare that they have no competing interests.

## Authors’ contributions

JGH: Design, data preparation, article preparation. SW: Data extraction, data analysis, article editing. Both authors read and approved the final manuscript.

## Author details

^1^vHerlev Hospital, University of Copenhagen, Graevlingestien 9, 2880, Bagsvaerd, Denmark. ^2^Research Unit of Clinical Epidemiology, Centre for National Clinical Databases – South, University of Southern Denmark and Odense University Hospital, Odense, Denmark.

## Pre-publication history

The pre-publication history for this paper can be accessed here:

http://www.biomedcentral.com/1471-2369/13/131/prepub
